# Existence of cholera outbreak, challenges, and way forward on public health interventions to control cholera outbreak in Guraghe Zones, southern Ethiopia, 2023

**DOI:** 10.3389/fpubh.2024.1355613

**Published:** 2024-05-27

**Authors:** Tamirat Melis Berhe, Yohannes Fikadu, Tadesse Sahle, Aklilu Habte Hailegebireal, Shamil Eanga, Temesgen Ketema, Shimelis Getu Wolde

**Affiliations:** ^1^Department of Public Health, College of Medicine and Health Science, Wolkite University, Wolkite, Ethiopia; ^2^Department of Midwifery, College of Medicine and Health Science, Wolkite University, Wolkite, Ethiopia; ^3^Department of Nursing, College of Medicine and Health Science, Wolkite University, Wolkite, Ethiopia; ^4^School of Public Health, College of Medicine and Health Science, Wachemo University, Hosanna, Ethiopia; ^5^Department of Anesthesia, College of Medicine and Health Science, Wolkite University, Wolkite, Ethiopia; ^6^Guraghe Zone Health Office, Disease Prevention and Control, Wolkite, Ethiopia; ^7^Department of Internal Medicine, College of Medicine and Health Science, Wolkite University, Wolkite, Ethiopia

**Keywords:** cholera outbreak, cholera eradication, mortality from cholera, challenges, elimination

## Abstract

**Introduction:**

In Ethiopia, despite major improvements seen in health service delivery system, the country continues to be significantly affected by cholera outbreaks. Cholera remains a significant public health problem among the vulnerable populations living in many resource-limited settings with poor access to safe and clean water and hygiene practices. Recurring cholera outbreaks are an indication of deprived water and sanitation conditions as well as weak health systems, contributing to the transmission and spread of the cholera infection.

**Objective:**

To assess the cholera outbreak, its challenges, and the way forward on public health interventions to solve the knowledge and health service delivery gaps related to cholera control in Guraghe Zone, Ethiopia, 2023.

**Methods:**

Active surveillance of the cholera outbreak was conducted in all kebeles and town administrative of Guraghe zone from 7/8/2023 to 30/10/2023. A total of 224 cholera cases were detected during the active surveillance method. Data obtained from Guraghe zone offices were exported to SPSS version 25 for additional analysis. The case fatality rate, incidence of the cases, and other descriptive variables were presented and described using figures and tables.

**Result:**

A total of 224 cholera cases were detected through an active surveillance system. In this study, the case fatality rate of cholera outbreak was 2.6%. To tackle the cholera outbreak, the Guraghe zone health office collaborated with other stakeholders to prepare four cholera treatment centers. The absence of OCV, inaccessible safe water, low latrine coverage, inappropriate utilization of latrines, and absence of cholera laboratory rapid diagnostics test in Guraghe Zone are barriers to tackling the outbreak.

**Conclusion:**

Ethiopia National Cholera Plan targeted eradicating cholera by 2030, 222 cholera outbreak occurred in Guraghe Zone, Ethiopia. To minimize and control cholera mortality rate oral cholera vaccinations should be employed in all areas of the region. Sustainable WASH measures should be guaranteed for the use of safe water and good hygiene practices. Early diagnosis and treatment should be initiated appropriately for those who are infected.

## Introduction

Cholera is an acute enteric disease caused by toxigenic *Vibrio cholera* (1). Though there are more than 206 *V. cholerae* species identified so far, only *V. cholerae* O1 and O139 serogroups cause *cholera* outbreak (2, 3). A study has shown that 20% of individuals infected with *cholera* develop acute watery diarrhea (4). Without timely and appropriate treatment, significant fluid and salt losses may lead to severe dehydration and mortality within hours. In untreated cases, the case-fatality rate could range from 30 to 50% (4).

In Asia and Africa, cholera continues to be an important public health concern that results in significant morbidity and mortality. While the majority of cases occur in low-income communities, cholera remains a significant global health concern. Both brief outbreaks and extended epidemics or pandemics of cholera can occur, and when left unchecked, the disease has disastrous effects on populations and their prospects. Investigation is essential to contain the outbreak, determine the risk factors that lead to it, and provide preventative and control measures (5).

It disproportionately impacts thousands of impoverished and vulnerable people in environments with limited resources (6, 7). Between 21,000 and 143,000 people die from cholera each year, and between 1.3 and 4.0 million new cases are reported globally. In South Asia and Africa alone, cholera cases account for 99% of cases worldwide (6). There are 120,652 cholera cases and 2,436 fatalities in 15 African nations (8). According to estimates, there are almost 40% more cases of cholera in West Africa, 32% more cases in East and the Horn of Africa, and 28% more cases in Central and Middle Africa. Central and middle Africa accounted for 43.4% of all deaths on the continent, almost 37.5% of deaths occurred in West Africa, and the remaining 19.1% in East Africa and the Horn of Africa (8). In Ethiopia 2,790 cholera cases were recorded during 2019–2020 of which 68 cholera cases were from southern Ethiopia (9). The cholera outbreak continues to pose a threat to public health and is a symbol of social injustice and regression. The WHO states that severe acute watery diarrhea, which can linger anywhere from 12 h to 5 days, is caused by drinking water or eating food contaminated with *V. cholerae* (6). Even if the infection rate is higher, most infected individuals are asymptomatic carriers (6). Therefore, because of problems, including poor reporting, limited epidemiological monitoring, and a lack of laboratory capacity, the incidence of cholera worldwide is underreported (6). This cholera outbreak is linked to these countries' where inadequate water, sanitation, and hygiene (WASH) infrastructure, and the disease is highly associated with low socioeconomic status, lack of access to safe drinking water, and poor hygiene practices (10). Controlling cholera and lowering mortality in a humanitarian setting requires a multimodal strategy that combines treatment, social mobilization, quick surveillance, oral cholera vaccines (OCV), and water, sanitation, and hygiene (WASH) (7, 11–13).

The Ethiopian government has also requested OCV doses from the Republic of Korea through the bilateral diplomatic channel to support large-scale reactive mass vaccination campaigns to control cholera outbreaks. The Ethiopian government has implemented strategies for cholera outbreak detection and containment, including preparing designated institutions for the treatment of cholera cases. This request was made to the WHO's Oral Cholera Vaccine (OCV) International Coordinating Group (ICG) for the emergency use of OCVs in 2019 (14–16). More recently, the Ethiopian government formally committed to a pathway for national cholera elimination and developed an extensive multi-sectoral national cholera control plan. As part of the Global Roadmap, the “Multi-sectorial Cholera Elimination Plan, Ethiopia 2021–2028” was authorized in 2021 and aims to eradicate cholera by 2030 (16). Despite different plans and strategies incorporated to eliminate cholera in Ethiopia, it still became a public health disease in Ethiopia and also in the world. The present study describes cholera outbreaks in Guraghe Zones, Southern Ethiopia and suggested to bridge the gaps in existing system to control cholera outbreaks.

## Methods

### Study setting and design

This study was conducted in the community of Guraghe Zone, southern Ethiopia. Guraghe Zone has 16 districts and eight administrative towns. It has 01 referral university hospital, 01 general hospital, 05 primary hospitals with 76 public health centers, and 413 health posts. All communities of the Guraghe zone are included in the surveillance system. But all cholera cases were obtained from only two three districts (Abeshge, sodo and Debub sodo districts). Descriptive epidemiology analysis conducted in residences of Guraghe zone from 7/8/2023 to 30/10/2023.

### Sampling procedure and data collection

Active surveillance of the cholera outbreak was conducted in all kebeles and town administrative of Guraghe zone (total of 275,778 households) from 7/8/2023 to 30/10/2023. From this cholera outbreak data, we assessed the challenges, control, and consequences of the cholera outbreak in the Guraghe Zone. A total of 224 cholera cases were detected during the active surveillance method from three Woreda (Abeshge, sodo and Debub sodo districts). Total population residing in this three district is 234,161. The cholera case detection rate was 224/234,161 = 96 cases per 100,000 population. The data collection tool was developed from a checklist of Ethiopian public health institutes for cholera outbreak risk assessment and checklist (17). It consists of socio-demographic characteristics, hygiene and sanitation activities, outcomes, and challenges of the study.

### Outbreak investigation

After one case of cholera occurred at a male patient of a daily laborer working on private farming land, the Rapid Response Team (RRT) was organized by the Guraghe Zone health office, and an outbreak investigation and control was initiated. A clinician, lab technician, communication specialist, epidemiologist, and environmental health specialist make up the RRT (18). To confirm reported cases of cholera, assess the scope of the outbreak, gather specimens for laboratory confirmation of *V. cholerae*, evaluate local capacity to respond to the outbreak, identify high-risk groups, look into the source of contamination, implement basic on-site control measures, provide emergency treatment supplies, and report the results of the outbreak investigation, the team conducts field assessments (18). From the register, the following information was gathered: name, age, sex, address, symptoms, date of illness onset, date treated, type of treatment given, and result of treatment (alive, dead, and referred), status of specimen collection, any risk-related information, and index case tracing. To determine any recent travel history, contacts with suspected cholera cases or/and sick people with diarrhea, attendance at a funeral recently (as well as the reason for the deceased's death), water sources (drinking, bathing, cleaning kitchen utensils), food consumption history, occupation, and any other risk factors for cholera transmission, interviews with the household members and neighbors of cholera cases required at the community level (18).

### Measures

#### Confirmed case of cholera

*Vibrio cholerae* O1 or O139 identified from the patient's feces confirms a case of cholera (5).

#### Case definition of cholera

The world health organization defines the case definition of cholera as the occurrence of acute watery diarrhea in a person aged 5 years or older living in an area where there is suspected or confirmed cholera, during an outbreak.

#### Diarrhea

As reported by the patient, mother, or caregiver, the passage of three or more loose/watery stools, or increasing frequency of stools in the 2 weeks preceding data collection.

#### Hand washing at all five critical times

A mother or caregiver judged to have fully exercised simple hand washing if she did so before eating, before preparing food, before feeding her kid, after cleaning the child, and after using the restroom. If she did not, she is regarded to have partially practiced the behavior (5).

#### Unimproved water source

When individuals drink water which is untreated with water treatment chemicals or that comes from a river, pond, well, or exposed spring (5).

#### Basic water supply

Access to sources of safe drinking water within a 30-min radius, such as rainwater collection, protected dug wells, public standpipes, boreholes, and household connections; also, disinfection of the home (5).

#### Basic sanitation

Access to better sanitation facilities, such as a pour-flush, simple pit, vented enhanced pit latrine, or connection to a septic system or public sewer.

### Data processing and analysis

Data obtained from the Guraghe zone office (in Excel format) was exported to SPSS version 25 for additional analysis. Calculations were made for the descriptive analysis using the percentage, frequency, and mean. The case fatality rate, incidences of the case, and other descriptive variables were presented and described using figures and tables.

## Results

### Descriptive epidemiology

The cholera outbreak originated on a private farm on 7th August 2023. It occurred at a male patient of daily laborer working in private farming land. The index case was reported to the Guraghe Zone office. He has a history of drinking contaminated water from the farmlands. He began to show acute watery diarrhea, vomiting, and dehydration with confirmation of *V. cholerae* from his stool sample. The number of cases reached a maximum on 8th August 2023.

### Socio-demographic characteristics of *Vibrio cholera* cases

The mean age of cholera cases was 24 ± 5 years. Of the total cholera cases, most 160 (71.4%) were males. The cholera cases were admitted and treated in three cholera treatment centers. Among the total cholera cases, 169 (75.4%) were farmers. Holy water accounts for 31% of sources of cholera cases (see Table 1).

**Table 1:** Socio-demographic characteristics of cholera patients in Guraghe Zone, Ethiopia, 2023.

**Characteristics**		**Frequency**	**Percentage (%)**
Age	<15	7	3.1
	15–30	151	67.4
	31–45	51	22.8
	>45	15	6.7
Sex	Male	160	71.4
	Female	64	28.6
Marital status	Single	124	55.3
	Married	100	44.7
Cholera treatment center	Borer CTC	105	46.9
	Bue Hospital	72	32.1
	Health center	47	21.0
Region	Addisabeba	13	5.8
	Southern region	181	80.8
	Oromiya	21	9.3
	Amhara	9	4.1
Occupation	Farmer	169	75.4
	Housewife	17	7.6
	Merchant	28	12.5
	Other	10	4.5
Source of cholera cases	Farmland	148	66.0
	Holy water	70	31.2
	community	6	2.8

Among the total cholera cases, 222 (99%) had watery diarrhea. Thirty one (14%) of cholera cases had no dehydration. Twenty-three (10.3%) of cholera cases use pipe water. More than half of the cholera cases 124 (54%) had no latrine (Table 2).

**Table 2:** Distribution of presenting clinical features, plan of rehydration management, the final status of cases, and household water and latrine status during the outbreak, Guraghe Zone, Ethiopia, 2023.

**Characteristics**		**Frequency**	**Percentage (%)**
Dehydration status	No	107	47.7
	Some	86	38.3
	Severe	31	14
Watery diarrhea	Yes	222	99
	No	2	1
Vomiting	Yes	187	83.4
	No	37	16.6
Water source	Pipe water	23	10.3
	River water	54	24.1
	Spring water	147	65.6
Contact history	Yes	125	55.8
	No	99	44.2
Travel History	Farmer	69	30.8
	Housewife	155	69.2
Latrine availability	Yes	100	44.6
	No	124	55.4
Outcome	Death	6	2.6
	Survive	218	97.4

#### Epidemic curve of cholera outbreak in Guraghe zone

**Figure 1:**
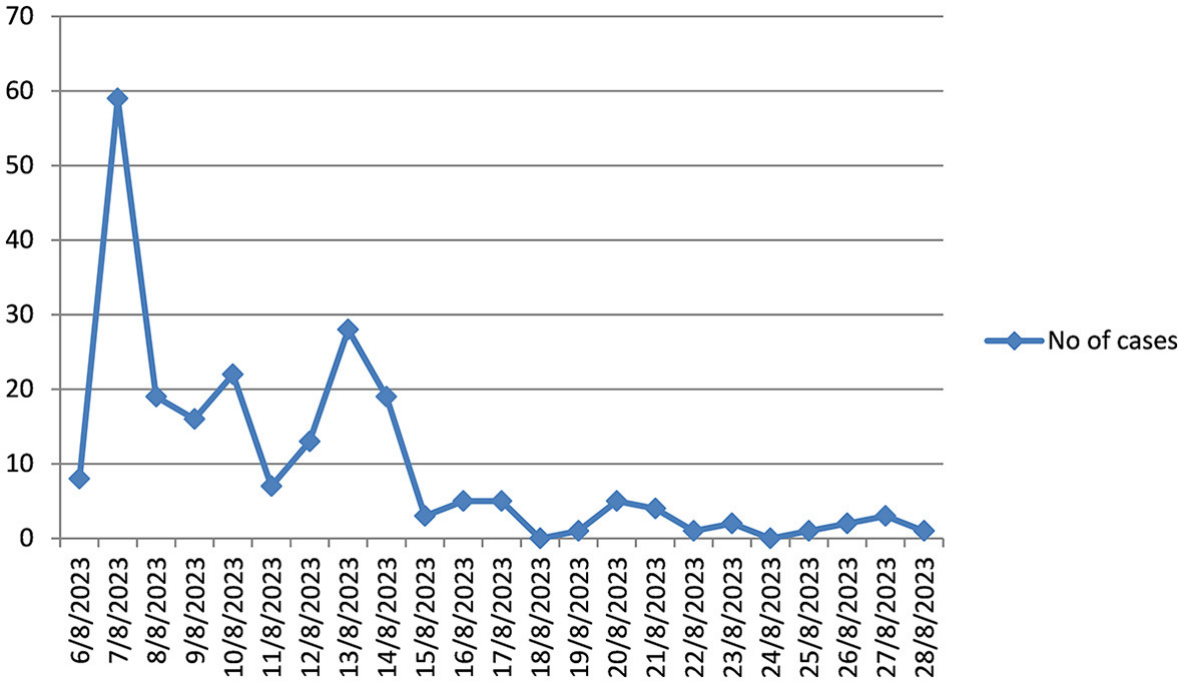
Incidence of cholera outbreak by date in Guraghe Zones, Southern Ethiopia, 2023.

The highest number of cholera cases was occurred in 7/8/2023. But starting from 29/8/2023; no cholera cases were detected for forty five consecutive dates (Figure 1).

#### Attack rates of cholera outbreak registered per reporting Woreda in Guraghe zone, Ethiopia, 2023

**Figure 2:**
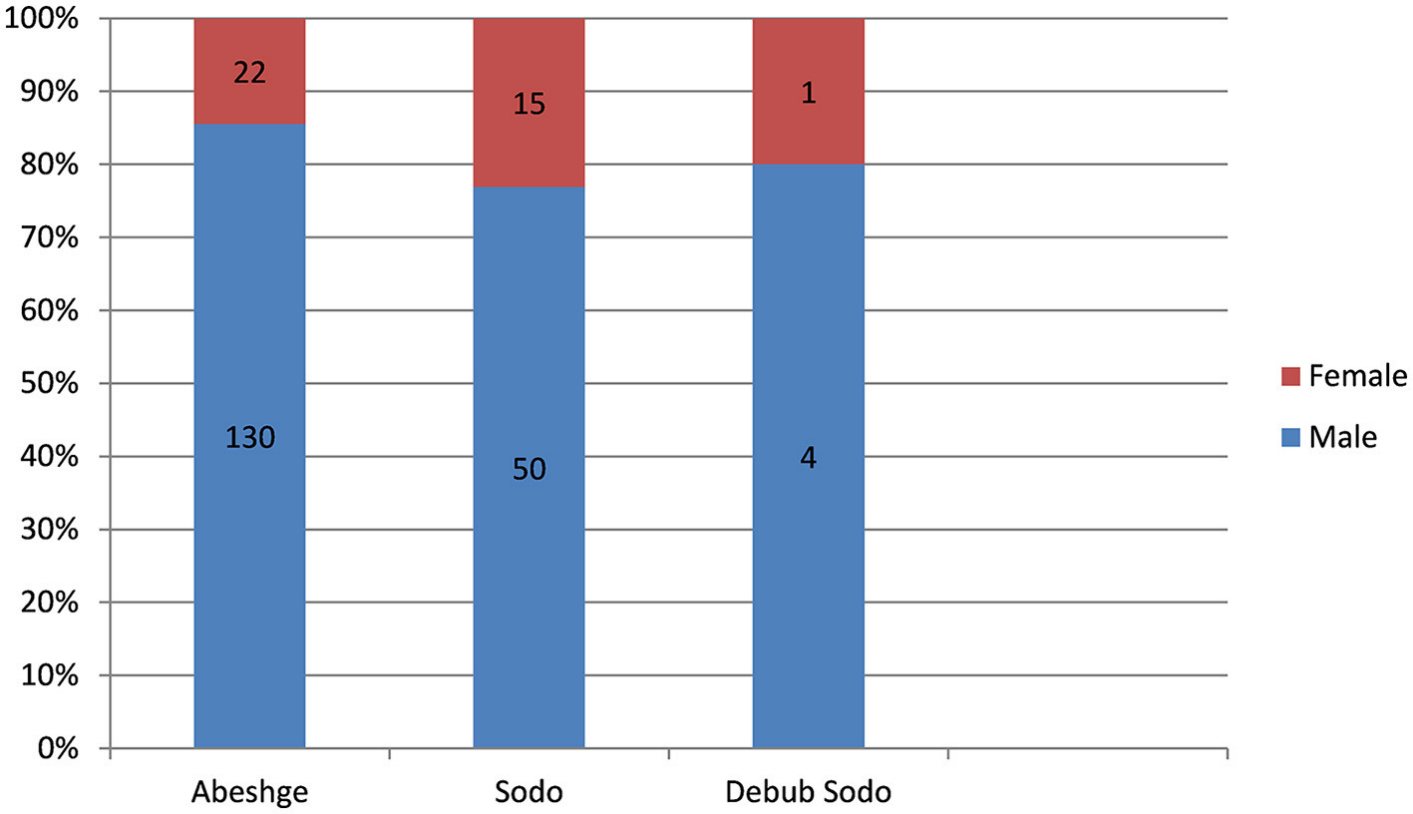
Attack rates of cholera outbreak by sex registered per reporting Woreda in Guraghe Zones, Ethiopia, 2023.

More than half 152 (67.8%), of the cholera cases were from Abeshge Woreda. From the Abeshge Woreda cholera cases, 130 (85.5%) of them were males (see Figure 2).

### Incidence of death with sex, age and occupation

Among the total cholera deaths, almost all of them 5 (83.3%) were males. Also among the total cholera deaths 5 (83.3%) were farmers (Table 3).

**Table 3:** Incidence of cholera death with age, sex and occupation in Guraghe Zone, Ethiopia, 2023.

**Variables**		**Cholera outcome**		**Total**
		**Alive**	**Died**	
Sex	Male	155	5	160
	Female	63	1	64
	Total	218	6	224
Occupation	Farmer	164	5	169
	Housewife	16	1	17
	Merchant	28	0	28
	Other	10	0	10
	Total	218	6	224

### Outcome of cholera cases

**Figure 3:**
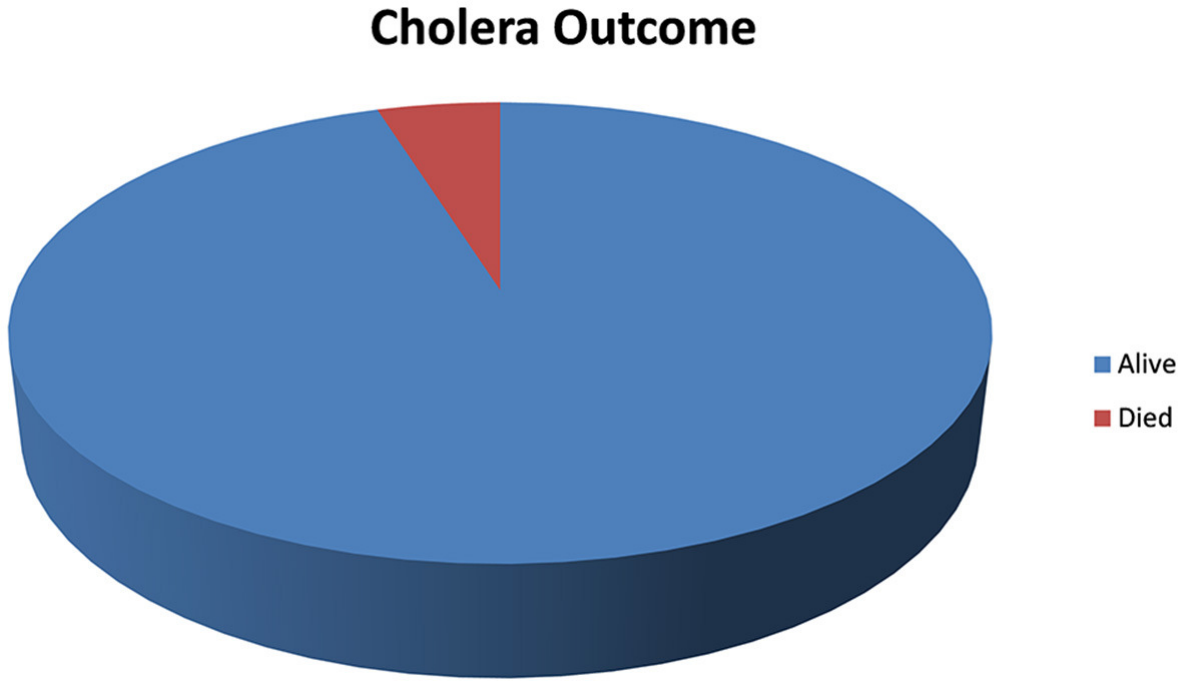
Outcome of cholera cases in Guraghe Zones, Southern Ethiopia, 2023.

A total of 224 cholera cases were diagnosed as cholera cases during the surveillance system. Six of them (8%) had died, while the remaining improved from their illness (see Figure 3).

### Cholera outbreak preparation and response activities

#### Coordination and collaboration

The Guraghe Zone prompted the establishment of multiple emergency coordination platforms at different levels of the health system to look into and contain the cholera outbreak. The establishment of an emergency command post at Guraghe Zone health office was among them. Every day, the command post convenes to discuss finished operations and give instructions on preparing for future emergencies and preventative measures in all kebeles. Additionally, an emergency response committee chaired by the Zonal PHEM processor and different stakeholders of Guraghe Zone participated in mitigating the outbreaks.

#### Case management

One of the measures implemented to control the outbreak was the establishment and standardization of oral rehydration points (ORPs) and cholera treatment centers (CTCs). To prevent the provision of health services for other health issues that may arise during disease outbreaks due to panic and an unplanned mobilization of human and other resources for emergency response, CTCs, and ORP sites were established and activated based on the number of cases received in health facilities.

#### Health education and social mobilization

Various health education campaigns utilizing diverse methods were implemented to inform the residents of the Guraghe Zone population about the cholera outbreak. A total of 275,778 households were visited. Every city and Kebele government employee received on-the-job training during the outbreak, and they took the duty of informing their neighbors and family members about the spread of cholera and how to prevent it. Loudspeakers are also used to spread health education to the homeless. Information for other community members was reached through media conferences, urban and rural health extension workers (HEWs), Women Development Army (WDA) leaders, and other community and religious organizations, such as churches and mosques.

#### Water, sanitation, and hygiene

Different WASH-related measures were taken to mitigate the transmission of the cholera outbreak. The team's main focus was testing and taking action in the area that is at risk for sources for cholera outbreaks. Seventy cases of cholera among a total of 224 cholera examined cases were from holly water site samples. All holly waters that are suspected of cholera sources were chlorinated. As part of the prevention and control effort, 3,813 private latrines and 10 community toilets were constructed. Water tankers were delivered and placed at various community locations where there was a water shortage and water quality was also monitored at different points and locations, such as sources, pipelines, and end lines. Water treatment and disinfection was conducted at 990 households using chlorine.

### Challenges faced during cholera outbreak prevention and control in Guraghe Zones

The cholera outbreak areas in the Guraghe zones (Abeshge and Bue woreda) were neighbors to the Wolayta and Hadiya zones, in which the cholera outbreak occurred before the occurrence of cholera in the Guraghe zone. Most of the cholera cases diagnosed in the Guraghe Zone came from the Hadiya and Wolayta Zone. There was no collaboration work with the health offices of these Zones. There was also poor hygiene and sanitation activity in Abeshge Woreda. Presence of weak social services (inaccessible Safe water and low coverage of latrines) increases the risk of cholera outbreaks.

The absence of the Oral cholera vaccine was another challenge during the outbreak. Also, a shortage of logistics (drugs, fluids, transportation, gloves, etc.) made the cholera outbreak investigation and control more difficult.

## Discussion

Ethiopia has created a National Cholera Elimination Plan, or NCP (2021–2028), by the Global Roadmap for the Control and Elimination of cholera. The objective is to achieve cholera cases (zero cases) in cholera hotspot areas by 2028 (19). This study assesses the existence of a cholera outbreak and how to investigate and control it in Ethiopia. A total of 224 cholera cases were detected during the active surveillance method from three Woreda (Abeshge, sodo and Debub sodo districts). However, previous study (that is conducted in Ethiopia from 2019 to 2020), showed that 2,790 cholera cases were recorded of which 68 cholera cases were from southern Ethiopia. This study showed that 224 cholera cases occurred in Guraghe zone (it is one of the 15 zones southern Ethiopia) (9). This might high number of cholera cases might be because of a high number of daily laborers entering from the nearby boundary (Wolayta Sodo, Goffa, and Hadiya Zone). These Zones were previously verified as the existence of a cholera outbreak. The other justification might be poor hygiene and sanitation cause progressive person to person transmission of *V. cholerae*. Total population residing in these three districts is 234,161. The cholera case detection rate was 124/234,161 = 53 cases per 100,000 populations. By 25 October 2022, about 273 cholera cases with nine deaths had been reported in east Bale (18) and 25 *V. cholerae* cases from Addis Ababa (20). The highest number of cholera cases was confirmed on the second day of existence of the outbreak.

This finding showed that the case fatality rate of *V. cholerae* was 2.6%. This finding is higher than the study conducted in Addis Ababa, Ethiopia (21). The justification might be the cholera treatment units formed in health centers. There were logistics and transportation problems in selected sites. The health center has no senior health professional (specialist). Also, the treatment center is far from the referral hospital which might lead to delays in early diagnosis and managing the cases. Poor case management in cholera treatment centers might be the other reason.

To tackle the cholera outbreak, the Guraghe zone health office collaborates with other stakeholders to play pivotal roles in controlling the outbreak. Rapid response team with different professions organized in the Guraghe Zone. The team prepared important resources, established the existence of a cholera outbreak, and verified the presence of the cholera outbreak. After verifying the presence of a cholera outbreak, they analyze to search for the precipitating factors to prevent and control the *V. cholerae* outbreak. Each day the team monitors and evaluates activities to tackle the cholera outbreak. They report all the activities done to mitigate the cholera outbreak to the regional health office.

Cholera treatment centers are prepared for admitting and treating cholera cases in important catchment areas. Important medical logistics were collected from different sources (Guraghe health office, private drug store, pharmacy, and private clinics). In addition to Guraghe Zone health office, Guraghe Zone Water and Irrigation, southern Regional Health bureaus, Guraghe Zone communication and agriculture office, and Guraghe Zone city administration had an important role in controlling cholera outbreak. In collaboration with these sectors, the Guraghe Zone health office identified 224 cases of *V. cholerae*. Awareness creation and community mobilization activities related to hand washing with soap before and after meals and latrine utilization, transmission of cholera through river water and uncooked fresh vegetables were conducted. A modeling study showed water treatment interventions at an adequate radius around cases used for cholera epidemic control (22).

Although different activities are done to tackle cholera outbreaks, an adequate OCV vaccination strategy for different cholera outbreak contexts and active case management at the community level are important to effectively prevent potential outbreaks, control transmissions, and reduce unreported deaths attributable to cholera. Several innovative OCV vaccination strategies have been introduced in a different cholera epidemic and endemic countries such as the case-area targeted interventions (CATIs) (23), ring vaccination (24, 25), self-administration of the second dose of OCV (26), and integration of WASH intervention delivery at health facilities with vaccination program (27). Recent systematic reviews and case studies on CATIs showed the approach used in 15 outbreaks in 12 countries, including the Democratic Republic of Congo, Haiti, Yemen, and Zimbabwe. The analysis showed interventions varied with WASH interventions more commonly implemented, and alert systems triggering interventions diverse from suspected cholera cases to culture-confirmed cases (28).

The other challenge was inaccessible safe water, low latrine coverage, and inappropriate utilization of latrines. Currently, the national sanitation coverage in Ethiopia reached around 57%, which results in more than 45 million people without access to appropriate sanitation facilities. These poor hygiene and sanitation practices precipitate cholera outbreak (26). The water supply and latrine coverage is particularly lower among households in lower socio-economic levels and remote areas, as well as some large crowd-gathering public sites such as marketplaces, bus stations, religious gathering sites, and even schools that can be potential cholera transmission hotspots (27). Only around 27% water supply coverage and 35% sanitation coverage are assessed in 45 woreda that have been identified as cholera hotspots (27).

The absence of laboratory rapid diagnostics test in Guraghe Zone prohibits the early detection and accurate diagnostics of *V. cholerae* and other causative pathogens associated with diarrheal diseases, which may also lead to inappropriate use of antibiotics which lead to poor management of *V. cholerae*. Therefore, there was a delay in early diagnosis and treatment of cholera cases because of the misclassification of cases. Lack of trained personnel both at the community and institutional level also compromises the investigation and control process. Most of the patients with cholera came from nearby boundaries (Hadiya, Wolayta, and Gofa Zones) which make it difficult to identify and treat index cases.

Going forward, a comprehensive monitoring of cholera case detection, healthcare facility, and laboratory capacities on cholera assessment, diagnostics, and reporting, ensuring OCV usage and various WASH projects according to available national guidelines. Lessons learnt from NCP development and roll-out in Ethiopia may serve as a reference for countries with similar public health agenda. Managing cross-border transmission of cholera cases especially with neighboring zones that share common water sources, corridors of transportation, and frequent movement of people remains an important area for multi-stakeholder policy dialogues and joint health research.

### Limitation of the study

This study used secondary data that could not find important variables like comparison (control group status which makes the analysis difficult). The sample size is not calculated because the study is conducted based on active surveillance that make the generalizability of the result more difficult.

## Conclusion

Although Ethiopia's National cholera plan targeted to eradicate cholera by 2030, 222 *V. cholerae* outbreaks occurred in Guraghe Zone, Ethiopia. A total of 224 cholera cases were detected through an active surveillance system that is high compared to previous study conducted in southern Ethiopia. The case fatality rate of *V. cholerae* in this study was 2.6%. A rapid response team with different professions was organized in the Guraghe Zone. The teams investigate and control the outbreak in collaboration with other sectors, prepare all important resources, establish the existence of an outbreak, and verify the presence of the cholera outbreak. After verifying the presence of an outbreak, they analyze for searching of the precipitating factors to prevent and control the *V. cholerae* outbreak. To control and minimize cholera mortality rates, oral cholera vaccinations should be employed in all areas of the region. Sustainable WASH measures should be guaranteed for the use of safe water, and good hygiene practices. For those who have already been infected, early diagnosis and treatment should be given appropriately.

## Data availability statement

The original contributions presented in the study are included in the article/supplementary material, further inquiries can be directed to the corresponding author.

## Author contributions

TB: Conceptualization, Data curation, Formal analysis, Funding acquisition, Investigation, Methodology, Project administration, Resources, Software, Supervision, Validation, Visualization, Writing – original draft, Writing – review & editing. YF: Resources, Software, Supervision, Validation, Visualization, Writing – original draft, Writing – review & editing, Conceptualization, Data curation, Formal analysis, Funding acquisition, Investigation, Methodology, Project administration. TS: Conceptualization, Data curation, Formal analysis, Funding acquisition, Investigation, Methodology, Project administration, Resources, Software, Supervision, Validation, Visualization, Writing – original draft, Writing – review & editing. AH: Conceptualization, Data curation, Formal analysis, Funding acquisition, Investigation, Methodology, Project administration, Resources, Software, Supervision, Validation, Visualization, Writing – original draft, Writing – review & editing. SE: Conceptualization, Data curation, Formal analysis, Funding acquisition, Investigation, Methodology, Project administration, Resources, Software, Supervision, Validation, Visualization, Writing – original draft, Writing – review & editing. TK: Conceptualization, Data curation, Formal analysis, Funding acquisition, Investigation, Methodology, Project administration, Resources, Software, Supervision, Validation, Visualization, Writing – original draft, Writing – review & editing. SW: Conceptualization, Data curation, Formal analysis, Funding acquisition, Investigation, Methodology, Project administration, Resources, Software, Supervision, Validation, Visualization, Writing – original draft, Writing – review & editing.
